# Ensemble Learning Models Based on Noninvasive Features for Type 2 Diabetes Screening: Model Development and Validation

**DOI:** 10.2196/15431

**Published:** 2020-06-18

**Authors:** Tianzhou Yang, Li Zhang, Liwei Yi, Huawei Feng, Shimeng Li, Haoyu Chen, Junfeng Zhu, Jian Zhao, Yingyue Zeng, Hongsheng Liu

**Affiliations:** 1 School of Life Science Liaoning University Shenyang China; 2 School of Information Liaoning University Shenyang China; 3 Research Center for Computer Simulating and Information Processing of Bio-macromolecules of Shenyang Liaoning University Shenyang China; 4 Engineering Laboratory for Molecular Simulation and Designing of Drug Molecules of Liaoning Shenyang China

**Keywords:** type 2 diabetes, screening, non-invasive attributes, machine learning

## Abstract

**Background:**

Early diabetes screening can effectively reduce the burden of disease. However, natural population–based screening projects require a large number of resources. With the emergence and development of machine learning, researchers have started to pursue more flexible and efficient methods to screen or predict type 2 diabetes.

**Objective:**

The aim of this study was to build prediction models based on the ensemble learning method for diabetes screening to further improve the health status of the population in a noninvasive and inexpensive manner.

**Methods:**

The dataset for building and evaluating the diabetes prediction model was extracted from the National Health and Nutrition Examination Survey from 2011-2016. After data cleaning and feature selection, the dataset was split into a training set (80%, 2011-2014), test set (20%, 2011-2014) and validation set (2015-2016). Three simple machine learning methods (linear discriminant analysis, support vector machine, and random forest) and easy ensemble methods were used to build diabetes prediction models. The performance of the models was evaluated through 5-fold cross-validation and external validation. The Delong test (2-sided) was used to test the performance differences between the models.

**Results:**

We selected 8057 observations and 12 attributes from the database. In the 5-fold cross-validation, the three simple methods yielded highly predictive performance models with areas under the curve (AUCs) over 0.800, wherein the ensemble methods significantly outperformed the simple methods. When we evaluated the models in the test set and validation set, the same trends were observed. The ensemble model of linear discriminant analysis yielded the best performance, with an AUC of 0.849, an accuracy of 0.730, a sensitivity of 0.819, and a specificity of 0.709 in the validation set.

**Conclusions:**

This study indicates that efficient screening using machine learning methods with noninvasive tests can be applied to a large population and achieve the objective of secondary prevention.

## Introduction

Diabetes is a heterogeneous metabolic disorder that is characterized by the presence of hyperglycemia due to impairment of insulin secretion, defective insulin action, or both [1]. The high blood glucose level caused by diabetes not only affects the heart, eyes, kidneys, and nerves but also is associated with increased rates of cancer, physical and cognitive disabilities [2-4], tuberculosis [5,6], and depression [7]; these conditions are associated with high health care costs [8,9]. For patients with type 2 diabetes, the risks of death and cardiovascular events are 2-4 times greater than in the general population [10]. Due to the aging population, lifestyle changes, and interrelated rapid unplanned urbanization, the prevalence of diabetes is quickly increasing worldwide [11]. According to the latest International Diabetes Federation Diabetes Atlas, there were approximately 420 million people aged 20-79 years with diabetes worldwide in 2017, and this number is expected to rise to 629 million in 2045. Furthermore, approximately 50% of diabetes patients are undiagnosed [12]. Patients with type 2 diabetes who are within target ranges for 5 risk factor variables, namely glycated hemoglobin levels, systolic and diastolic blood pressure, albuminuria, smoking, and low-density lipoprotein cholesterol levels, appear to have little or no excess risk of death, myocardial infarction, or stroke compared with the general population [13]. Therefore, developing an appropriate method to screen people without clinical symptoms is necessary and practical; such a screening method could reduce health care costs and patient mortality and improve patients’ quality of life through earlier clinic-based management.

Generally, traditional screening projects are based on studies in epidemiology, such as the ADDITION trial study [14] and the Ely study [15]. These screening studies cost hundreds of thousands of dollars and require the collaboration of many people. With the emergence and development of machine learning, researchers have started to pursue more flexible and efficient methods to screen or predict type 2 diabetes. Han et al [16] trained a type 2 diabetes diagnosis model with features mainly consisting of blood tests such as hemoglobin A1_c_ and total cholesterol, yielding a precision of 0.942 and a recall of 0.939. Maniruzzaman et al [17,18] trained a type 2 diabetes prediction model using Pima Indian data with plasma glucose features; they obtained an accuracy of 81.97% and an area under the curve (AUC) of 0.93. A machine learning–based framework was also developed to identify patients with type 2 diabetes in the clinic with electronic health records, showing an AUC of 0.98 with more than 110 clinical features [19]. Zou et al [20] used principal component analysis and minimum redundancy maximum relevance to reduce the dimensionality and achieve the best accuracy in their model (0.81) in addition to using fasting blood sugar as the main feature. Many of the abovementioned studies achieved high prediction performance with blood tests; however, none of them used only noninvasive attributes to predict type 2 diabetes. Chung et al [21] developed a model to screen prediabetes using support vector machines with only noninvasive features, such as age, sex, and family history of diabetes, and they obtained an AUC of 0.76 in the external test data; however, further exploration and optimization are needed to improve type 2 diabetes screening models that only use noninvasive features.

To better screen potential patients with type 2 diabetes, further delay disease progression, control relative complications, and improve human health, in this paper, type 2 diabetes screening machine learning models and conforming easy ensemble models were built that require only an individual noninvasive test, combined with data from body measurements and questionnaires, to predict type 2 diabetes based on the National Health and Nutrition Examination Survey (NHANES) database, thus avoiding blood tests and clinic visits. Inexpensive screening of people who have type 2 diabetes without obvious symptoms may lead to secondary prevention.

## Methods

### Analysis

The data were analyzed with R version 3.3.1 for Linux with the R packages dplyr, caret (Classification And REgression Training) [22], randomForest [23], pROC [24], e1071 [25], gplots, unbalanced [26], epiDisplay, and MASS. The Delong test for 2 correlated receiver operating characteristic (ROC) curves was used to determine the effects of the easy ensemble methods; a *P* value <0.05 was considered significant (2-sided). The work protocol consisted of 5 steps: data cleaning, sample selection, chosen features, model training, and validation.

### Data

**Figure 1:**
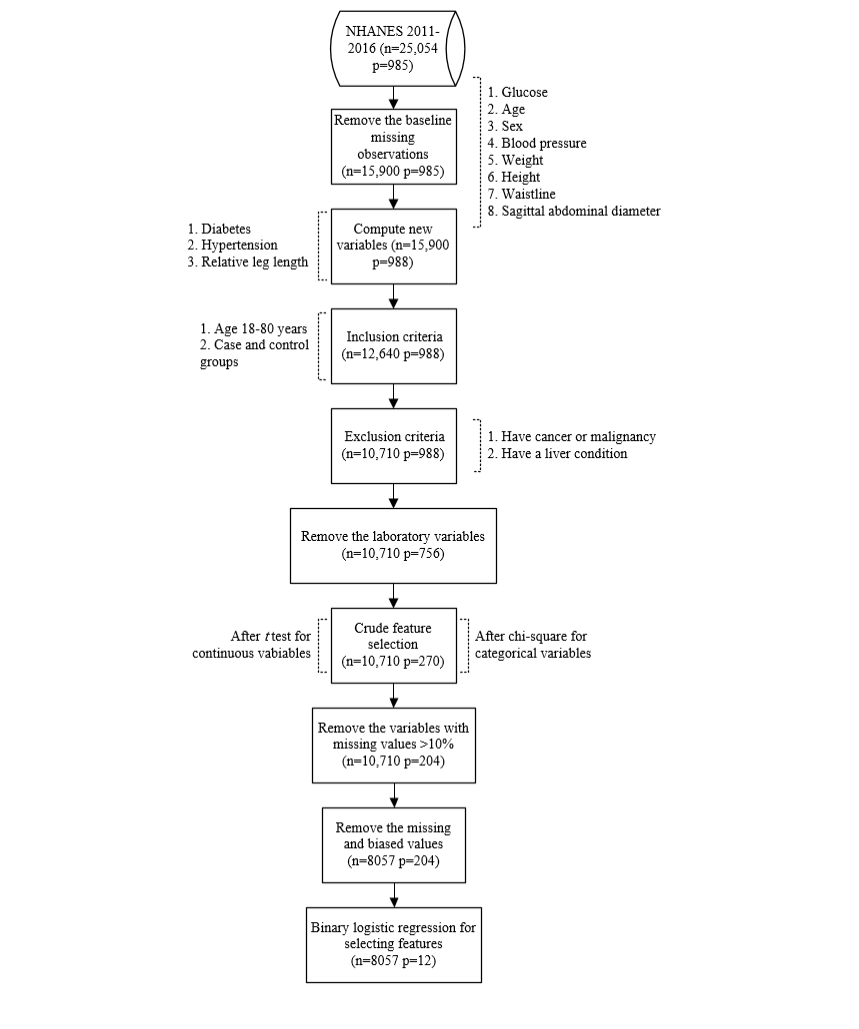
The data cleaning and feature selection process. Note that the feature selection process was run only in the NHANES 2011-2014 dataset. n: number of cases. p: number of features.

The data were obtained from the NHANES database. The detailed steps of data cleaning and feature selection are shown in Figure 1. First, before all the NHANES data were processed, the database contained 25,054 samples from 2011 to 2016 with 985 features. Second, data samples with missing observations for baseline variables, such as blood glucose, age, sex, height, and weight, were removed. Third, 3 new variables were computed, namely diabetes (whether a person has diabetes: 1=yes, 0=no), hypertension (whether a person has hypertension: 1=yes, 0=no) and relative leg length. The case group was defined as having fasting blood glucose levels ≥7.0 millimoles per liter, and the fasting blood glucose levels in the control group were <6.1 mmol/L [1]. Hypertension was defined according to the American Heart Association criteria as systolic blood pressure ≥130 millimeters of mercury or diastolic blood pressure ≥80 mm Hg obtained on more than 2 occasions [27]. The relative leg length was the ratio of the upper leg length to the height multiplied by 100 [28]. Fourth and fifth, we set the inclusion and exclusion criteria to control for bias. The inclusion criteria were as follows: patients aged 18-80 years from the case and control groups. The following exclusion criteria were employed: patients with cancer, due to the positive association between hyperglycemia and cancer [29], and patients with liver conditions, because liver conditions can also influence blood glucose levels [30]. These individuals were excluded because they are traditionally asymptomatic and their blood glucose levels are not representative of the study population. After the data processing steps (1-5), 10,710 observations and 988 features without type 2 diabetes were left for analysis.

### Feature Selection

The selection of features is one of the most critical steps in model building. Thus, additional feature selection steps were taken. First, because only noninvasive features were used, the laboratory variables were deleted, and 756 features were left. Secondly, we used the *t* test to select continuous variables and the chi-square test to select the categorical variables for crude feature selection with *P*<.05; this resulted in 270 remaining features. Third, the variables whose missing values were greater than 10% were removed, leaving 204 features. Fourth, the missing and biased values (including answers in the questionnaire such as “refused” and “don’t know”) were deleted, leaving 8057 samples. Finally, forward conditional logistic regression was employed to further filter the features that were selected in the former steps with *P*<.05 only in the NHANES 2011-2014 dataset. After the feature selection process, 12 features remained. We separated the final dataset into three parts: the training set (80%, 2011-2014) with 3582 negative and 664 positive observations, the test set (20%, 2011-2014) with 895 negative and 165 positive observations, and the external validation set (2015-2016) with 2244 negative and 507 positive observations; the whole 2011-2014 data set was randomly divided into the training set and test set using the createDataPartition function in the caret package [22].

### Machine Learning and the Easy Ensemble Method

In this study, binary logistic regression was used to select the risk factors for diabetes, and the linear discriminant analysis, random forest, and support vector machine methods as well as their ensemble methods were developed to classify the case and control groups according to the selected features. The linear discriminant analysis structure was based on the lda function of the R package MASS, the support vector machine structure was based on the svm function of the R package e1071, and the random forest structure was based on the rf function of the R package randomForest. The parameter adjustments of the support vector machine and random forest were applied with the R package caret. We used 80% of the 2011-2014 NHANES data for model training under 100 repeated 5-fold cross-validations. The remaining 20% of the 2011-2014 NHANES data were used as the test set, and the 2015-2016 NHANES data were reserved as the validation set for performance measurement.

#### Logistic Regression

As an extension of linear regression, logistic regression is a commonly used method to obtain the risk or protection factors for disease in epidemiology [31,32]. According to the experimental design, this logic function was divided into unconditional and conditional logistic regressions; according to the type of dependent variables, it was divided into binary logistic regression and multiple logistic regression. The logistic function is an effective method for classification problems and gives the odds ratio (OR) of the significance variable according to the dependent variable.

In this study, binary unconditional logistic regression was used to select the risk factors for or relative features of diabetes. In the logistic regression, the 204 attributes chosen from the *t* test and chi-square test were considered as the independent variables, and whether a person has diabetes was the dependent variable. Twelve features were left.

#### Linear Discriminant Analysis

Linear discriminant analysis was first introduced by Fisher [33] in 1936 to address taxonomic problems. Generally, it is a combination of analysis of variance and regression analysis. Linear discriminant analysis is based on the theory of transformation from high dimensions to low dimensions. As a classification algorithm, its theoretical basis is that the protection points of each type of data are as close to each other as possible, while the distance between different kinds of data are as far apart as possible. In this case, the classification was based on whether a person has diabetes. Therefore, the linear discriminant analysis reduced the 12 features to the 1(k–1, k=2) dimension to discriminate patients with diabetes.

#### Random Forest

Random forest, which is based on decision trees [34], is a well-known ensemble learning method that uses the bagging method [35]. The basic theory of the bagging method is as follows: assuming a dataset contains N observations, for example, 100 subsets can be extracted wherein every subset comprises n (n=N) observations that were sampled randomly with replacement from the original dataset, and 100 base classifiers can be built with these 100 subsets to vote for the classification of every sample in the dataset. The decision trees are the base classifier in the bagging method in the random forest. This basic algorithm can be considered as a single tree model with if-then structures. Each decision tree of the RF yields its own classification outcome and “vote,” and the average of all the results is the final taxonomy.

The caret package in R was applied to search for the best parameter in the random forest with 5-fold cross-validation repeated 100 times. The number of trees was 500, and the best number of variables randomly sampled as candidates at each split was 4 after the parameter selection.

#### Support Vector Machine

Support vector machines [36] are among the most popular supervised learning techniques in the machine learning field. A support vector machine reflects the data to a higher-dimensional space with a kernel function. The classification mission relies on the training data, which are called support vectors. For general 2-class problems, the observations are determined by a hyperplane with the maximizing margin through the nearest support vectors.

In this study, the radial basis kernel was chosen. The caret package of R was also used to match the parameter with the best AUC performance in the support vector machine model with 5-fold cross-validation repeated 100 times. The optimal cost and gamma parameter values obtained for the model were 0.137 and 0.012, respectively.

#### Easy Ensemble Method

Type 2 diabetes screening is an unbalanced problem because there are fewer patients than healthy individuals. To address the unbalanced issue, we employed the easy ensemble method [37]. In short, we randomly sampled the same number of all positive observations from the negative observations and made the two groups correspond to a minor dataset in the train set. We then repeated the above step 100 times to generate 100 minor datasets. Next, we built 100 same-method models based on these datasets. Furthermore, for 5-fold cross-validation, the prevalence probability of every sample was averaged by these 100 models in every validation for both the test set and validation set.

### Model Evaluation

In this article, we used the ROC curve, AUC, sensitivity, specificity, accuracy, and positive predictive value (PPV) to measure the performance of the models. The cutoff value was selected based on the maximal value of the Youden index [38] in the training set.

## Results

After the data cleaning and feature selection process, the dataset included 8057 cases that were divided into three sets: 80% of the NHANES 2011-2014 data for the training set, 20% of the NHANES 2011-2014 data for the test set, and the NHANES 2015-2016 data for the validation set. After crude feature selection with the *t* test and chi-square test in the 2011-2014 NHANES dataset, logistic regression analysis was further performed to assess the related factors of type 2 diabetes; this process ensures that there will be no overfitting or generalization of the model for future patients. The 12 selected factors are shown in Table 1.

**Table 1:** Factors associated with diabetes used to build the models.

Feature	Crude^a^ OR^b^ (95% CI)	Adjusted^c^ OR (95% CI)	*P* value
Age	1.05 (1.05-1.06)	1.05 (1.04-1.06)	<.001
Sex	0.82 (0.70-0.97)	0.62 (0.50-0.76)	<.001
Waistline	1.04 (1.03-1.05)	0.99 (0.97-1.01)	.27
Sagittal abdominal diameter	1.20 (1.18-1.22)	1.16 (1.09-1.24)	<.001
Relative leg length	0.70 (0.66-0.74)	0.85 (0.79-0.91)	<.001
60 second pulse	1.02 (1.01-1.02)	1.02 (1.01-1.03)	<.001
Smoking	0.74 (0.63-0.88)	1.13 (0.92-1.38)	.26
Alcohol	1.43 (1.19-1.72)	1.31 (1.04-1.66)	.02
Hypertension	3.26 (2.72-3.90)	1.02 (0.82-1.27)	.86
Family history	0.28 (0.24-0.34)	0.32 (0.26-0.39)	<.001
General health condition	2.05 (1.88-2.24)	1.59 (1.44-1.76)	<.001
Control or loss of weight	0.42 (0.35-0.51)	0.55 (0.44-0.69)	<.001

^a^Crude: 1-way logistic regression.

^b^OR: odds radio.

^c^Adjusted: multiple logistic regression.

The risk of having type 2 diabetes increases with increased age (95% CI 1.04-1.06, *P*<.001), sagittal abdominal diameter (95% CI 1.09-1.24, *P*<.001), pulse (95% CI 1.01-1.03, *P*<.001), and alcohol use (95% CI 1.04-1.66, *P*=.02) as well as poorer general health condition (95% CI 1.44-1.76, *P*<.001). In contrast, female sex, longer relative leg length, lack of type 2 diabetes family history, and control of weight are the protection factors of type 2 diabetes (95% CI 0.50-0.76, 0.79-0.91, 0.26-0.39, and 0.44-0.69, respectively; *P*<.001 in all cases). We built three different models using linear discriminant analysis, random forest, and support vector machine methods to determine type 2 diabetes risk using the training set with these noninvasive tests. Afterward, the test set and external validation set were used to measure the predictive ability of the models.

**Figure 2:**
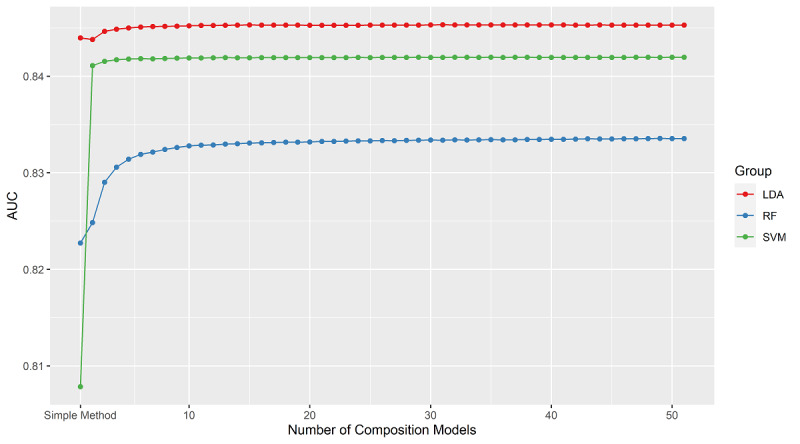
Comparison of the top 50 models with the easy ensemble method and the simple method with different machine learning methods and 5-fold cross-validation in the training set. AUC: area under the curve. LDA: linear discriminant analysis. RF: random forest. SVM: support vector machine.

We generated six models with three different machine learning methods as well as corresponding ensemble methods in the training set. The 5-fold cross-validation results in Table 2 show that the linear discriminant analysis method yielded the best AUC compared with the random forest and support vector machine methods not only with the simple methods but also with the easy ensemble methods. However, the ensemble method improvements in the different methods are in the order of support vector machine > random forest > linear discriminant analysis. In 5-fold cross-validation, the simple linear discriminant analysis method showed 0.844 AUC, 74.1% sensitivity, 79.5% specificity, 78.7% accuracy, and 40.2% PPV; the ensemble linear discriminant analysis method showed 0.845 AUC, 79.7% sensitivity, 73.5% specificity, 74.5% accuracy, and 35.8% PPV. The simple random forest method showed 0.823 AUC, 86.2% sensitivity, 61.2% specificity, 65.1% accuracy, and 29.2% PPV; its ensemble method showed 0.834 AUC, 78.4% sensitivity, 73.2% specificity, 74.0% accuracy, and 35.2% PPV. The simple support vector machine method showed 0.808 AUC, 69.2% sensitivity, 81.1% specificity, 79.2% accuracy, and 40.5% PPV; the ensemble support vector machine method showed 0.842 AUC, 78.7% sensitivity, 74.8% specificity, 75.4% accuracy, and 36.7% PPV. The line graph in Figure 2 shows that the AUC improved with accumulation of the models, and the values remained stable after the composition of approximately 10 models.

**Table 2:** Average results (SD) of the 5-fold cross-validation of the models in the training set.

Method		AUC^a^	Sensitivity	Specificity	Accuracy	PPV^b^
**Simple methods**						
	Linear discriminant analysis	0.844 (0.016)	0.741 (0.035)	0.795 (0.015)	0.787 (0.013)	0.402 (0.020)
	Random forest	0.823 (0.016)	0.862 (0.029)	0.612 (0.019)	0.651 (0.015)	0.292 (0.011)
	Support vector machine	0.808 (0.015)	0.692 (0.035)	0.811 (0.017)	0.792 (0.014)	0.405 (0.023)
**Ensemble methods**						
	EE^c^ linear discriminant analysis	0.845 (0.016)	0.797 (0.032)	0.735 (0.016)	0.745 (0.014)	0.358 (0.017)
	EE random forest	0.834 (0.016)	0.784 (0.033)	0.732 (0.016)	0.740 (0.014)	0.352 (0.016)
	EE support vector machine	0.842 (0.016)	0.787 (0.034)	0.748 (0.017)	0.754 (0.014)	0.367 (0.018)

^a^AUC: area under the curve.

^b^PPV: positive predictive value.

^c^EE: easy ensemble method.

The 5-fold cross-validation indicated that the different models show reliable capability. Similarly, the AUCs of the developed models range from 0.810-0.850 in the test and validation datasets, indicating their stability and extensibility for predicting the risk of new patients with type 2 diabetes. Furthermore, when considering the performance of the easy ensemble methods in the test set (Table 3), these methods appeared to predict type 2 diabetes more efficiently than the other methods. For the random forest and support vector machine methods, the easy ensemble methods provided significantly better AUC values than the respective simple methods (absolute AUC improvement 0.014, z=3.062, *P*=.002 and 0.07, z=5.010, *P*<.001, respectively), as determined by the Delong test for two correlated ROC curves (2-sided). However, the LDA improvement was not significant (z=1.252, *P*=.21) according to the Delong test. In the validation set (Table 3), we found a similar pattern. The easy ensemble methods improved the overall predictive performance by 0.004 (z=2.734, *P*=.006) for linear discriminant analysis, 0.008 (z=2.991, *P*=.002) for random forest, and 0.037 (z=5.908, *P*<.001) for support vector machine.

The results indicate that the ensemble methods can be used to screen large populations for type 2 diabetes based on their significantly improved performance in the tests for the random forest and support vector machine methods and in the external validation set for the linear discriminant analysis, random forest, and support vector machine methods. For better and easier application of type 2 diabetes screening, a screening website based on the ensemble method has been established [39].

**Table 3:** Performance of the simple and ensemble methods in the text and validation sets.

Method			AUC^a^	Sensitivity	Specificity	Accuracy	PPV^b^
**Test set**							
	**Simple methods**						
		Linear discriminant analysis	0.864	0.697	0.829	0.808	0.429
		Random forest	0.836	0.830	0.648	0.676	0.303
		Support vector machine	0.796	0.630	0.864	0.827	0.460
	**Ensemble methods**						
		EE^c^ linear discriminant analysis	0.867	0.758	0.777	0.774	0.385
		EE random forest	0.850	0.776	0.770	0.771	0.383
		EE support vector machine	0.861	0.752	0.783	0.778	0.390
**Validation set**							
	**Simple methods**						
		Linear discriminant analysis	0.846	0.759	0.762	0.761	0.418
		Random forest	0.828	0.888	0.594	0.648	0.331
		Support vector machine	0.811	0.720	0.789	0.776	0.435
	**Ensemble methods**						
		EE^c^ linear discriminant analysis	0.849	0.819	0.709	0.730	0.389
		EE random forest	0.836	0.813	0.713	0.731	0.390
		EE support vector machine	0.848	0.824	0.714	0.734	0.394

^a^AUC: area under the curve.

^b^PPV: positive predictive value.

^c^EE: easy ensemble method.

## Discussion

### Comparison With Prior Work

The results of one analysis predicted that the world ranking of the number of years of life lost due to diabetes will increase from 15th to 7th [40] by 2040. The fact that type 2 diabetes damages health conditions deserves special attention. In this article, we generated type 2 diabetes screening models and applied them to a large population. Although some researchers [16-20] have studied machine learning models for screening and predicting type 2 diabetes, most of their studies focused on improving performance by selecting many features, such as blood test results, instead of considering the practical significance of cost and flexibility. In contrast, we used a noninvasive test covering demographic factors, body measurements, and questionnaire variables to build our models; this addresses the shortcomings of using invasive tests. Jai Won Chung et al [21] also adopted noninvasive features to predict prediabetes, including age, gender, family history of diabetes, hypertension, alcohol intake, BMI, smoking status, waist circumference, and physical activity; they obtained a best AUC of 0.76 in the external test data. However, the attributes they chose were relatively traditional compared with those chosen in this study; in addition, the similarities between prediabetes and healthy cases can result in lower AUC values. The validation of our models indicates that body measurements and questionnaire questions can be used to predict whether a person has type 2 diabetes. In the case of further effects resulting from high blood sugar conditions, the models can be used to screen the identified people.

### Principal Results

In the feature selection process in this study, traditional analyses such as the *t* test, chi-square test, and binary logistic regression were used. We extracted unusual attributes related to type 2 diabetes, such as sagittal abdominal diameter, relative leg length, and heart rate, which were proven to be significant in similar studies [28,41,42], in addition to some common risk factors, such as age, sex, alcohol use, and family history [43,44]. Among these features, relative leg length was an interesting clue to type 2 diabetes that has not previously been used in type 2 diabetes prediction; this feature was selected by *t* test and forward conditional logistic regression. Epidemiological studies from various settings indicate that humans with shorter legs relative to their stature have higher risk for type 2 diabetes [28]. Relative leg length can be easily determined and has a strong correlation with type 2 diabetes; therefore, it may be a useful new attribute in model building or epidemiology research. With increasing adoption of this feature, our model will be more accurate and dependable.

Reliable type 2 diabetes screening models based on noninvasive tests and machine learning algorithms were established and validated in this study. All the easy ensemble methods yielded higher predictive performance (AUC≥0.85 and AUC≥0.83, respectively) in the test set and validation set than the simple methods, indicating the efficiency of the ensemble methods. Screening models based on population are always an unbalanced problem, with more negative samples and fewer positive samples in the whole dataset. In other words, the learning ability of the models is not satisfied by the positive samples. We randomly matched a negative sample for every positive sample and generated 100 base models. This type of repeated learning from the positive samples may improve the results of the models. In addition to AUC, the application of the ensemble can increase the steadiness of the performance; this was exhibited by other measurements, such as sensitivity, specificity, accuracy, and PPV. Compared with different machine learning methods, the ensemble method improvement is limited; this suggests that the dataset and features are more essential. In recent research, the results show that individuals with screen-detected type 2 diabetes were diagnosed earlier and had better outcomes than those who were clinically detected with regard to all-cause mortality, cerebrovascular disease, renal disease, and retinopathy [45]. In addition to earlier ordinal treatment, Ej et al [46] introduced a method to recover the function of islets by diet control. Regardless of treatment, quality of life improvement and decreased disease burden are important.

### Limitations

There are several limitations of our research project. The World Health Organization definition of diabetes is inferior to proper diagnosis by an experienced physician; also, we cannot clearly separate type 1 diabetes from type 2 diabetes, which would cause bias because of their different epidemiological attributes. After removing the baseline missing values and executing the inclusion and exclusion criteria, there were 10,710 samples in the entire database. Additionally, 2653 missing and biased values were removed. The proportion of patients with diabetes to patients without diabetes is approximately 1:5; therefore, the increased amount of abandoned diabetes data may reduce the predictive ability of the model. Reproducibility remains doubtful given the variable demographics of the different datasets. Only a study using noninvasive features to screen for diabetes can minimize the impact of demographic changes such as those considered in large population health studies and nutrition surveys. The best PPV was only 0.435 in the validation set; this indicates that only approximately 40% of true positive samples from the people detected positively by these models were patients with type 2 diabetes. A higher false-positive value increases the financial expenses of the health care system in the beginning; however, this type of screening program can improve the overall health of the population, and earlier diagnosis can decrease the disease burden, ultimately decreasing health care expenses related to diabetes. On one hand, although the easy ensemble method [37] applied here addresses the unbalanced problem in one sense, more positive observations may yield better performance; on the other hand, the building of type 2 diabetes screening models is always an imbalanced problem when screening patients with type 2 diabetes from a large population. Therefore, we cannot solve the unbalanced problem completely. After considering all the other possible biases influencing the performance of the models, the key point is to further explore and optimize the unbalanced problem.

### Conclusions

Accurate models with low-cost variables based on NHANES data for screening type 2 diabetes were established; the models performed better with the application of ensemble methods. The use of NHANES data by the models ensured a sufficient sample size, and the models can be a tool to determine the health conditions of people who were not included in the survey. Compared with prior literature, this study has certain advantages, such as noninvasive features and reliable model performance. However, we still obtained low PPV results for the unbalanced problem and could not completely solve the missing value problem. Furthermore, we can not only optimize the method by incorporating more quality data from medical schools but can also combine our study with a cohort study to achieve primary prevention.

## Abbreviations

AUC: area under the curve
caret: Classification And REgression Training
NHANES: National Health and Nutrition Examination Survey
OR: odds ratio
PPV: positive predictive value
ROC: receiver operating characteristic
